# Immune checkpoint TIM-3 defines hyperactivated NK cells and predicts fatal outcome in severe fever with thrombocytopenia syndrome

**DOI:** 10.1371/journal.pntd.0013928

**Published:** 2026-01-16

**Authors:** Xiaohan Qian, Yi Zhang, Ziling Cheng, Haolin Song, Lingtong Huang, Bei Jia, Jie Li, Jie Wang, Wei Wu

**Affiliations:** 1 State Key Laboratory for Diagnosis and Treatment of Infectious Diseases, National Clinical Research Center for Infectious Diseases, National Medical Center for Infectious Diseases, Collaborative Innovation Center for Diagnosis and Treatment of Infectious Diseases, The First Affiliated Hospital, Zhejiang University School of Medicine, Hangzhou, China; 2 Department of Laboratory Medicine, the First Affiliated Hospital, Zhejiang University School of Medicine, Hangzhou, China; 3 Department of Critical Care Units, the First Affiliated Hospital, Zhejiang University School of Medicine, Hangzhou, China; 4 Department of Infectious Diseases, Nanjing Drum Tower Hospital, Affiliated Hospital of Medical School, Nanjing University, Nanjing, Jiangsu, China; Seoul National University College of Medicine, KOREA, REPUBLIC OF

## Abstract

**Background:**

Severe fever with thrombocytopenia syndrome (SFTS) is a viral hemorrhagic fever with high mortality, primarily driven by immune dysregulation. This study aimed to characterize the expression profile of TIM-3 expression on peripheral NK cells in SFTS, explore the role of TIM-3 ⁺ NK cells in disease progression, and evaluate soluble TIM-3 (sTIM-3) and Galectin-9 (sGalectin-9) as prognostic biomarkers.

**Methods:**

Public single-cell RNA sequencing (scRNA-seq) datasets were analyzed using weighted gene co-expression network analysis (WGCNA). Peripheral blood samples from 21 SFTS patients and 14 healthy donors collected at the First Affiliated Hospital, Zhejiang University School of Medicine, were analyzed by flow cytometry, functional assays, and serological assessments. NK cell cytotoxicity was assessed by granzyme B, perforin, IFN-γ, and TNF-α production. Serum sTIM-3 and sGalectin-9 were measured by Cytometric Bead Array.

**Results:**

TIM-3 ⁺ NK cells were significantly increased in SFTS patients, especially in fatal cases. WGCNA identified *HAVCR2* (encoding TIM-3) as a mortality-associated hub gene. TIM-3 ⁺ NK cells exhibited enhanced granzyme B and perforin expression, while TIM-3 blockade significantly reduced IFN-γ and TNF-α production. Elevated sTIM-3 and sGalectin-9 levels were correlated with fatal outcomes.

**Conclusions:**

Activated TIM-3 ⁺ NK cells were associated with fatal outcomes in SFTS. TIM-3 ⁺ NK cell proportion, sTIM-3, and sGalectin-9 may serve as novel prognostic biomarkers and therapeutic targets.

## Author summary

While previous studies have described immune dysregulation in SFTS (including cytokine storms, T cell exhaustion, and impaired B cell responses), the role of NK cells remained unclear. This study employs single-cell RNA sequencing, flow cytometry, and serological analysis to define a high-resolution landscape of circulating NK cell heterogeneity in SFTS. We identify significant upregulation of TIM-3 (HAVCR2) on NK cells, particularly in fatal cases, associated with hyperactivated cytotoxic function. Furthermore, we demonstrate that circulating TIM-3 ⁺ NK cells, soluble TIM-3 (sTIM-3), and Galectin-9 (sGal-9) serve as potential prognostic biomarkers for disease severity and outcome. Our findings indicate that TIM-3 ⁺ NK cells contribute to SFTS immunopathogenesis through hyperactivation. Serum Galectin-9 represent novel biomarkers for early risk stratification. These results also highlight the modulation of the TIM-3 immune checkpoint pathway as a promising therapeutic strategy to curb excessive immune activation in acute viral infections like SFTS.

## Introduction

Severe Fever with Thrombocytopenia Syndrome (SFTS) is an acute infectious disease caused by the *Dabie bandavirus*, which belongs to the *Phenuiviridae* family, *Bandavirus* genus of viruses [1]. The pathogen is transmitted primarily through tick bites, and can also be spread through contact with the blood or bodily fluids of infected patients. The virus was first identified in China in 2009 and has since been reported in other Asian countries, including South Korea, Japan, Vietnam and Myanmar, with reported mortality rates ranging from 12% to 50% [2–4]. SFTS, particularly in severe and fatal cases, manifests as a viral hemorrhagic fever characterized by thrombocytopenia, lymphopenia, systemic inflammation, coagulopathy, and multi-organ failure [5,6]. Despite its clinical severity and high mortality, the treatment for SFTS remains largely supportive [3]. The pathogenesis of SFTS is characterized by vigorous viral replication and an excessive host immune response, both of which synergistically contribute to pathological damage and clinical deterioration [4]. A dysregulated immune response, including cytokine storm [7–9], impaired innate immunity, defective antiviral T cell function [10], and suboptimal humoral responses [11,12], plays a pivotal role in the progression to fatal outcomes. However, the mechanisms underlying severe disease development remain incompletely understood, highlighting the urgent need to identify prognostic biomarkers and therapeutic targets.

Natural Killer (NK) cells are crucial components of the innate immune system and play essential roles in both cytotoxicity and immune regulation. NK cells eliminate virus-infected cells primarily through cytotoxic mechanisms, including perforin- and granzyme-mediated killing, and by secreting cytokines such as IFN-γ and TNF-α, thereby providing critical protection against viral infections such as Epstein-Barr virus and cytomegalovirus [13]. Beyond their antiviral roles, NK cells may also exacerbate inflammation through cytokine storms. In patients with COVID-19, enhanced NK cell activation and cytotoxicity have been associated with increased disease severity [14].

T cell immunoglobulin and mucin-domain containing-3 (TIM-3) is an inhibitory immune checkpoint glycoprotein initially identified on IFN-γ-producing CD4⁺ and CD8 ⁺ T cells [15], and later found to be expressed on various immune cells, including NK cells, monocytes, macrophages, and regulatory T cells [16]. Emerging evidence suggests that TIM-3 plays a multifaceted role in NK cell biology, influencing disease progression, immune regulation, and therapeutic responsiveness. Cytokine stimulation, such as by IL-15, IL-12, or IL-18, can induce TIM-3 expression, implicating it as a marker of NK cell activation and maturation [17]. However, the functional impact of TIM-3 appears to be context-dependent. In chronic infections and malignancies, TIM-3 expression is often associated with impaired NK cell cytotoxicity and IFN-γ production, suggestive of NK cell exhaustion [18,19]. Conversely, in certain settings such as head and neck squamous cell carcinoma (HNSCC), high TIM-3 expression correlates with enhanced NK cell cytotoxicity, and this enhancement can be counteracted by Galectin-9-mediated inhibition [20].

SFTS is characterized by profound immune dysregulation, yet the dynamics of NK cell phenotypes and functions during acute infection and in critically ill patients remain incompletely understood [21,22]. Here, we aimed to elucidate how NK cell dysregulation contributes to SFTS disease progression and to define the role of TIM-3 in modulating NK cell functions. In this study, we demonstrate that TIM-3 expression on NK cells correlates with fatal outcomes, and that TIM-3 ⁺ NK cells in the peripheral blood of SFTS patients exhibit enhanced effector potential, potentially contributing to exacerbated immunopathology and poor clinical outcomes.

## Methods

### Ethics statement

Written informed consent was obtained from all participants in accordance with the ethical principles of the Declaration of Helsinki and the Belmont Report. The study protocol was approved by the Ethics Committee of the First Affiliated Hospital of Zhejiang University School of Medicine (Approval Number: 2022-119) and the Ethics Committee of Nanjing Drum Tower Hospital (approval number: 2022-238-02).

### Single-cell RNA-seq data processing

We obstained scRNA data from previously published scRNA-seq datasets GSE175499 [4].The Cell Ranger toolkit V3.1.0 provided by 10x Genomics was applied to align reads and generate the gene-cell unique molecular identifier (UMI) matrix against the reference genome GRCh38. Then the matrix data was loaded into Seurat for initial processing. The output CellRanger expression profile matrix was loaded into Seurat (version 4.1.0) for filtering of low-quality cells from scRNA-seq data, and the filtered data was downscaled and clustered. For each cell, we quantified the number of genes kept high-quality cells with 500–8000 genes detected and no more than 25% of mitochondrial gene counts. Cells were projected into 2D space using UMAP.

### Cell type annotation and cluster marker identification

The Seurat FindAllMarkers function was employed with default settings to identify marker genes for each cell cluster. Clusters were annotated using classical markers for specific cell types. The annotation results were further validated by comparison with the Annotation of Cell Types [23] and manual curation. Although unsupervised clustering is widely used for identifying cell populations in scRNA-seq data analysis, we observed that NK cell identification inevitably included other cell types. Therefore, we first isolated potential NK cells based on the positive expression of *NCAM1* or *KLRF1* (expression > 0) and the negative expression of CD3 genes (including *CD3D*, *CD3G*, and *CD3E*) [24]. Following this, clustering was performed using Seurat (v4.1.0) with standard preprocessing steps, including FindVariableFeatures, ScaleData, RunPCA, RunUMAP, FindNeighbors, and FindClusters.

### DEG identification and functional pathway enrichment

Differentially expressed genes (DEGs) were identified using the FindMarkers function in Seurat with default parameters (logfc.threshold = 0.26, test.use = “bimod”, min.pct = 0.1). Functional enrichment analysis of the identified gene sets was performed using the enrichPathway function from the clusterProfiler package (v3.15.2) [25] based on the Kyoto Encyclopedia of Genes and Genomes (KEGG) pathway and Gene Ontology (GO) database. Enriched pathways were identified based on adjusted *p*-values < 0.05, with the false discovery rate estimated using the Benjamini-Hochberg method.

### Cell-cell interaction analysis and trajectory analysis

CellChat [26] was performed by integrating cellular gene expression and cell type information to explore cell interactions and determine the mechanism of communication molecules at the single-cell level. The single-cell pseudotime trajectory analysis of single cells was conducted with the Monocle3 package [27].

### RNA velocity analysis

RNA velocity was estimated using the Python package velocyto (v0.2.2) [28] to re-count spliced and unspliced UMIs from the single-cell transcriptomic data. Subsequently, velocity computation was performed with the stochastic modeling approach implemented in the scvelo functions within the velociraptor package (v3.18) in R. To ensure consistency, calculations were restricted to the set of genes previously used for data integration. For visualization, RNA velocity pseudotime was projected onto the UMAP embedding to illustrate dynamic transitions in NK cell states.

### Weighted gene co-expression network analysis (WGCNA)

Normalized expression matrices of NK cells were extracted from the Seurat object using the subset function in the Seurat package. Weighted gene co-expression network analysis (WGCNA) was then performed following the standard workflow to identify modules associated with SFTS fatal-related outcomes [29]. All genes were included to comprehensively screen for potential prognostic signatures. A soft-thresholding power of 2 was selected based on the scale-free topology fit index, as it achieved R^2^ = 0.85 while intersecting the high-value threshold line in the scale independence plot. Modules were constructed based on topological overlap matrix dissimilarity, with a minimum module size set to 50 genes.

### Identification of modules and hub genes

To identify modules significantly correlated with disease prognosis, Pearson correlation analyses were performed between module eigengenes (MEs) and clinical trait data. Within modules showing significant associations with mortality, gene significance (GS), defined as the correlation between individual genes and the trait, and module membership (MM), representing the correlation between genes and their corresponding MEs, were assessed. The module showing the strongest trait correlation and the highest GS–MM association was designated as the key module. The co-expression network of this prognostically relevant blue module (965 genes) was exported to Cytoscape for visualization and clustering [30]. Using the MCODE algorithm [31], two major gene clusters were identified. To prioritize hub genes, both clusters were further ranked by Maximal Clique Centrality (MCC) using the cytoHubba plugin in Cytoscape [32].

### Patients and clinical samples

This prospective cohort study enrolled adult patients (≥18 years) with confirmed SFTS, who were admitted to the First Affiliated Hospital of Zhejiang University School of Medicine between January 1, 2023, and December 31, 2024. In accordance with national treatment guidelines issued by the Chinese Ministry of Health, 21 patients receiving standard antiviral and supportive therapies were included. SFTS was confirmed by positive polymerase chain reaction detection of Severe fever with thrombocytopenia syndrome virus (SFTSV) in peripheral blood samples. Individuals co-infected with hepatitis B virus, hepatitis C virus, or human immunodeficiency virus-1, or those with clinical or laboratory evidence of concurrent bacterial or fungal infections, were excluded. A control group comprising 14 age- and sex-matched healthy blood donors was included for comparative analysis. Detailed demographic, clinical, and laboratory results were collected from electronic medical records, with baseline characteristics summarized in Table 1. An independent validation cohort consisting of 104 laboratory-confirmed SFTS patients from Nanjing Drum Tower Hospital was included, and detailed demographic and clinical information is summarized in S3 Table.

**Table 1:** Baseline characteristics of SFTS patients.

	Control	SFTS		*P* Value
	(N = 14)	Recovered (N = 17)	Deceased (N = 4)	
**Demography**				
Age	68 (62 - 74)	62 (56 - 67)	77 (72 - 82)	0.092
Male Sex (n,%)	8 (57.1)	8 (47.1)	3 (75.0)	0.710
SpO_2_/FiO_2_	471.4 (466.7 - 476.2)	337.9 (303.0 - 471.4)	307.6 (129.3 - 467.9)	**0.008**
GCS score at admission	15.0 (15.0 - 15.0)	15.0 (15.0 - 15.0)	12.0 (9.5 - 14.3)	**0.002**
Days from symptom onset to hospital admission (day)	NA	7.0 (5.0 - 7.0)	7.5 (4.8 - 10.5)	0.716
Hospitalization period (day)	NA	14.0 (14.0 - 18.0)	5.5 (3.3 - 7.5)	**0.003**
**Comorbidity**				
Hypertension (n,%)	7 (50.0)	6 (35.3)	3 (75.0)	0.270
Diabetes (n,%)	2 (14.3)	3 (17.6)	0 (0.0)	0.848
Cancer (n,%)	1 (7.1)	3 (17.6)	2 (50.0)	0.136
CCI (median [IQR])	3.0 (2.0 - 3.0)	3.0 (2.0-3.0)	6.0 (5.5 - 6.8)	**0.009**
**Laboratory parameters**				
WBC (10^9^/L)	6.4 (5.5 - 7.4)	5.2 (2.5 - 6.2)	6.1 (4.5 - 7.7)	0.111
PLT (10^9^/L)	242.0 (212.8 - 309.0)	72.0 (42.0 - 128.0)	47.5 (29.8 - 69.3)	**<0.001**
Lymphocyte (%)	36.4 (30.5 - 39.6)	17.7 (12.5 - 36.7)	8.7 (7.8 - 14.4)	**0.014**
Monocyte (%)	8.0 (6.9 - 9.6)	7.6 (4.4 - 13.5)	3.2 (1.7 - 7.6)	0.355
ALT (U/L)	18.0 (11.5 - 29.5)	66.0 (27.0 - 110.0)	104.0 (47.5 - 212.0)	**<0.001**
AST (U/L)	22.5 (17.0 - 29.5)	106.0 (38.0 - 207.0)	357.0 (218.5 - 753.3)	**<0.001**
Cr (μmol/L)	80.5 (71.8 - 87.8)	72.0 (53.0 - 81.0)	116.5 (92.0 - 159.0)	**0.003**
Viral load (10^5^copies/ml)	NA	0.0 (0.0 - 0.0)	9.2 (5.1 - 19.9)	**0.002**

Data are presented as median [interquartile range] or n (%), as appropriate. Statistical analysis was performed using the Kruskal–Wallis H test and Chi-square test. For multiple comparisons, significance was adjusted using the Bonferroni correction. GCS, Glasgow Coma Scale; CCI, Charlson comorbidity index; WBC, white blood cell count; PLT, platelet count; ALT, alanine aminotransferase; AST, aspartate aminotransferase; Cr, creatinine; FiO₂, fraction of inspired oxygen; SpO₂, peripheral oxygen saturation; SFTS, severe fever with thrombocytopenia syndrome; NA, not available.

### Sample collection and processing

Peripheral blood samples were collected from SFTS patients within 24 hours of hospital admission. Age- and sex-matched blood samples from healthy donors were obtained at the time of enrollment. All blood samples were processed within 4 hours of collection. Peripheral blood mononuclear cells (PBMCs) were isolated via density gradient centrifugation using Ficoll-Paque Plus (Dakewe Biotech, China) according to the manufacturer’s instructions.

### Flow cytometry

Peripheral blood samples were processed for surface and intracellular flow cytometric analysis using standard protocols. The gating strategy for NK cells is shown in Fig 4A. Briefly, leukocytes were identified based on CD45 (APC; BD Bioscience) expression and side scatter (SSC) characteristics. NK cells were defined as CD45^+^CD3^−^CD56^+^CD16^+^ (BD Bioscience). For surface staining, freshly isolated PBMCs (5 × 10⁵ cells per sample) were incubated with fluorochrome-conjugated antibodies against CD3 (APC-Cy7), CD56 (PE), and TIM-3 (BV421; BioLegend) at 4 °C for 20 min. Isotype-matched controls were included in all experiments. Red blood cells were lysed using TQ-Prep reagents (Beckman Coulter) according to the manufacturer’s protocol.

**Fig 1:**
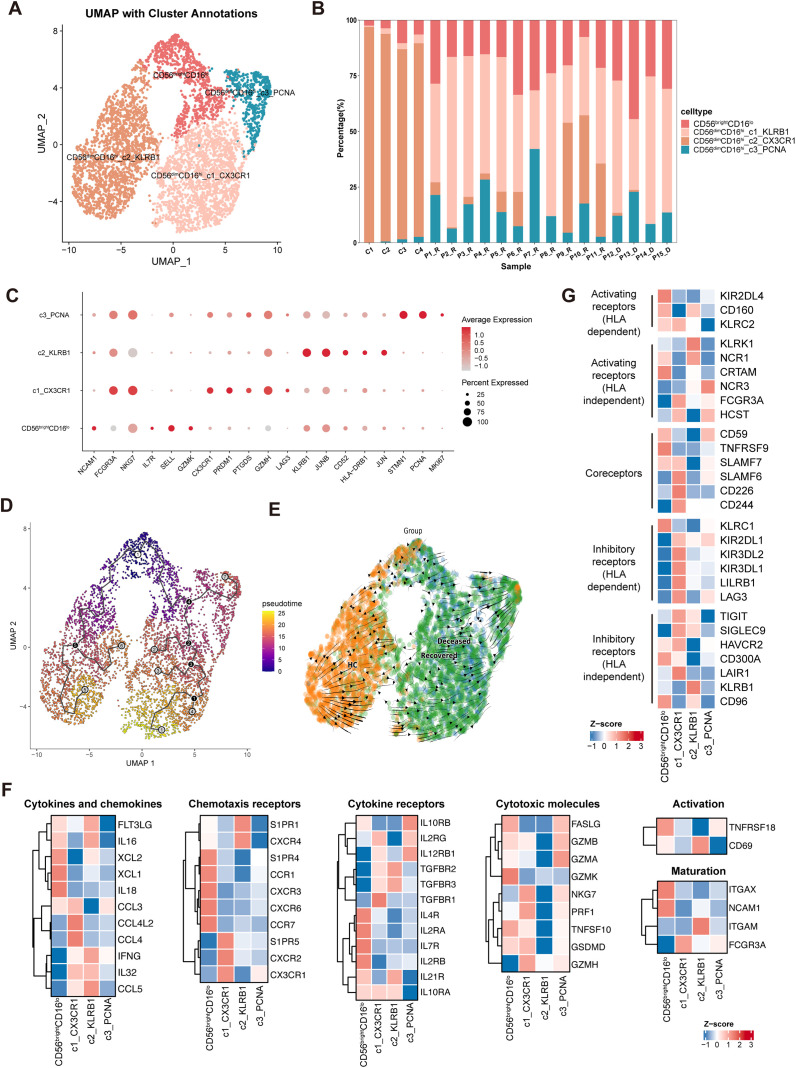
The immune landscape of circulating NK cells in SFTS patients. **(A)** UMAP plot showing NK cell subpopulations identified from peripheral blood of SFTS patients and healthy controls. **(B)** Stacked bar plot illustrating the distribution of NK cell clusters across individual samples. **(C)** Heatmap displaying representative marker genes defining each NK cell cluster. **(D)** Pseudotime trajectory analysis of NK cell differentiation states across clusters. **(E)** RNA velocity analysis indicating the directionality and dynamics of NK cell state transitions. **(F)** Heatmap of NK cell clusters categorized based on canonical functional markers. **(G)** Expression heatmap of activating and inhibitory NK cell receptors across identified clusters.

**Fig 2:**
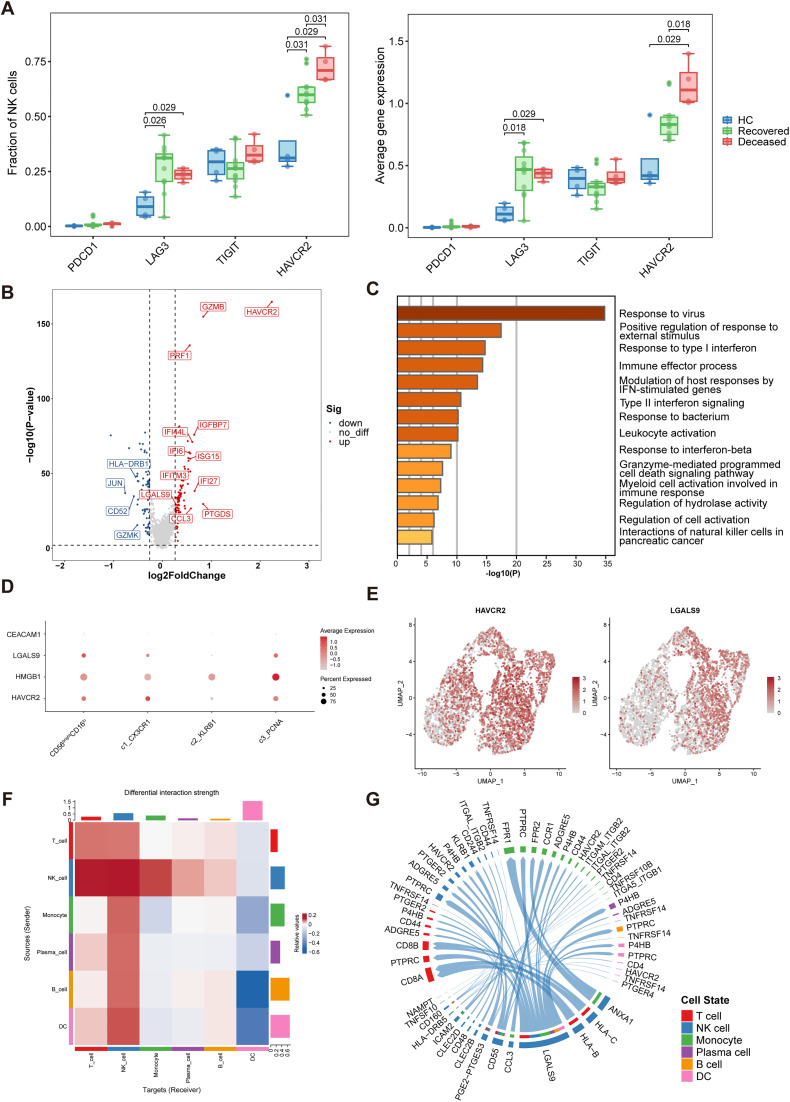
Upregulation of TIM-3 expression in circulating NK cells from SFTS patients. **(A)** Proportions and expression levels of immune checkpoint molecules in circulating NK cells. **(B)** Volcano plot showing differentially expressed genes between TIM-3^+^ and TIM-3^-^ NK cells. **(C)** Enriched pathways identified by Metascape comparing TIM-3^+^ and TIM-3^-^ NK cells. **(D)** Expression levels of *HAVCR2* (encoding TIM-3) and its ligand genes (*CEACAM1*, *HMGB1*, *LGALS9*) across NK cell subsets. **(E)** Feature plots displaying the expression of *HAVCR2* and *LGALS9* in NK cells. **(F)** Cell-cell communication analysis by CellChat showing enhanced interactions between NK cells and other immune populations in fatal SFTS compared to recovered cases. **(G)** Chord diagram illustrating *LGALS9*-associated signaling pathways enriched in NK cells from fatal cases, including the *LGALS9*–*HAVCR2* axis. Box plots show the median and interquartile range. Statistical analysis was performed using the Wilcoxon rank-sum test. DC, dendritic cell; NK, natural killer cell; SFTS, severe fever with thrombocytopenia syndrome.

**Fig 3:**
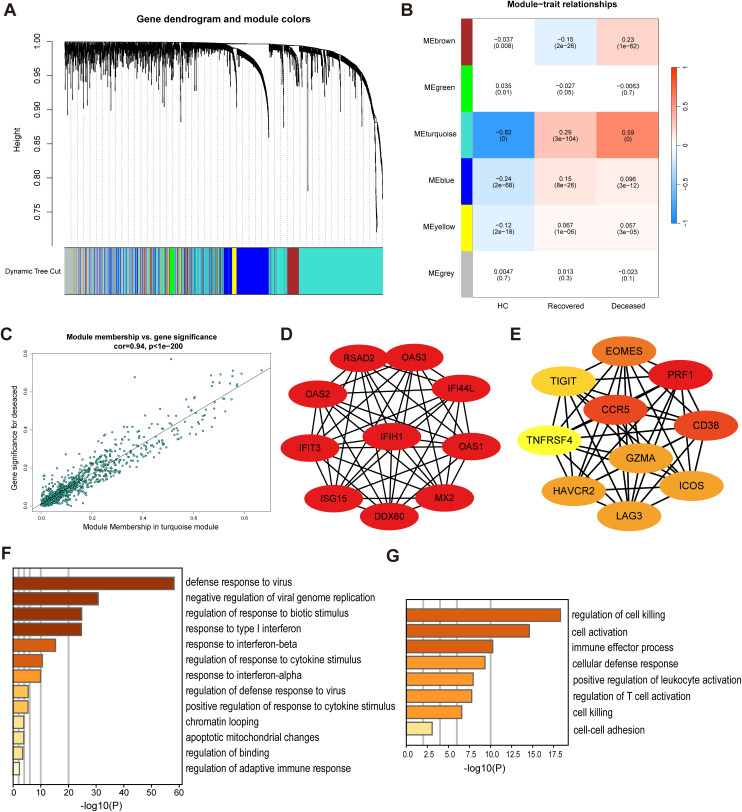
WGCNA identifies prognosis-associated hub genes in SFTS. **(A)** Gene clustering dendrogram constructed by weighted gene co-expression network analysis (WGCNA). **(B)** Module–trait relationships showing the turquoise module as significantly correlated with clinical outcome. **(C)** Correlation between gene significance and module membership within the turquoise module for the fatal outcome phenotype. **(D)** Top 10 hub genes from the most highly connected cluster, forming an interferon-related co-expression network. **(E)** Top 10 hub genes from the second highly connected cluster, including *HAVCR2*, within a prognosis-associated regulatory network. **(F)** Gene Ontology (GO) enrichment analysis of the first cluster hub genes reveals immune response and interferon signaling pathways. **(G)** GO enrichment analysis of the second cluster hub genes highlights cytotoxic pathways.

For cytokines and CD107a detection, PBMCs were stimulated with 50 ng/ml phorbol 12-myristate 13-acetate (PMA) and 500 ng/ml ionomycin (both from Sigma-Aldrich) for 6 h in the presence of brefeldin A (BD Biosciences). For intracellular staining, cells were fixed and permeabilized using the Cytofix/Cytoperm kit (BD Biosciences), followed by staining with antibodies against granzyme B (BV510), perforin (APC), IFN-γ (PE), and TNF-α (APC) at 4 °C for 20 min. A separate staining panel was used for CD107a (APC) to avoid fluorochrome conflict with TNF-α. Data were acquired using a FACSCanto II flow cytometer (BD Biosciences), with a minimum of 50,000 events collected per sample, and analyzed using FlowJo software (Tree Star, version 10.2).

### TIM-3 blockade assay

To assess the functional effect of TIM-3 signaling on NK cell activation, freshly isolated PBMCs from SFTS patients were incubated in RPMI 1640 medium supplemented with 10% fetal bovine serum and anti-TIM-3 blocking antibody (BioLegend, 10 μg/ml) or matched isotype control for 30 minutes at 37°C. After 18–24 hours of incubation, cells were harvested for subsequent functional analyses. PBMCs were stimulated with 50 ng/mL PMA and 500 ng/mL ionomycin (Sigma-Aldrich) for 3 hours in the presence of brefeldin A (BD Biosciences) for the detection of cytotoxic molecules granzyme B and perforin during TIM-3 block assay. For cytokines and CD107a detection, PBMCs were stimulated as described above. Including intracellular cytokine staining and CD107a detection by flow cytometry, or culture supernatants were collected for cytokine quantification via ELISA or Cytometric Bead Array (CBA). Intracellular cytokine staining for IFN-γ and TNF-α was performed as described above.

### NK cell isolation

Human NK cells were isolated from peripheral blood mononuclear cells (PBMCs) by negative selection using the human NK Cell Isolation Kit (Miltenyi Biotec, Bergisch Gladbach, Germany), according to the manufacturer’s instructions. In brief, PBMCs were resuspended in cold degassed buffer (PBS pH 7.2, 0.5% BSA, 2 mM EDTA) and incubated first with a biotin-conjugated antibody cocktail against non-NK cell lineages for 5 minutes, followed by a second incubation with anti-biotin MicroBeads for 10 minutes, with both steps performed at 4°C. The magnetically labeled cell suspension was then applied to a pre-rinsed LS column placed in a separator. The unlabeled, untouched NK cells were collected in the flow-through as the enriched fraction. The purity of the isolated NK cells, defined as CD3 ⁻ CD56 ⁺ lymphocytes, was consistently verified to be > 95% by flow cytometric analysis post-isolation.

### Measurement of cytokines in NK cell culture supernatants

The cytokine production capacity of NK cells was evaluated under different stimulation conditions. Briefly, isolated NK cells were seeded in 96-well plates at a density of 1 × 10⁶ cells/mL. The following experimental conditions were established: unstimulated control, stimulated control, and a group pre-treated with a TIM-3 blocking antibody prior to stimulation for 30 minutes. Cells were stimulated with a cell stimulation cocktail (Invitrogen) for 12 hours, after which supernatants were collected or purified NK cells were incubated for 16 hours with IL-12 (1 ng/mL) and IL-18 (10 ng/mL; both from GenScript). Cells were washed in PBS, treated with 10ug/mL anti-Tim-3 mAb and incubated for 30 minutes at 37°C. Recombinant human Gal-9 (rhGal-9; GenScript) and incubated for 6 hours. The concentrations of IFN-γ and other cytokines in the culture supernatants were quantified using commercial ELISA kits (R&D system) or CBA Kits (Cellgene Biotech), according to the manufacturers’ instructions.

### Serum measurement of soluble TIM-3, Galectin-9, TNF-α and IFN-γ

Serum concentrations of soluble TIM-3 (sTIM-3) and Galectin-9 (sGalectin-9) were measured using Human Tim-3 and Galectin-9 Cytometric Bead Array (CBA) kits (BioLegend, San Diego, CA, USA), while serum levels of interferon-gamma (IFN-γ) and tumor necrosis factor-alpha (TNF-α) were measured using cytokine CBA kits (Cellgene Biotech, Shanghai, China); all assays were performed according to the manufacturer’s instructions. Serum was collected from whole blood by centrifugation at 1500 g for 10 minutes and stored at –80°C prior to use.

### Statistical analysis

Unless otherwise specified in the figure or table legends, statistical analyses were conducted using GraphPad Prism version 7.03 (GraphPad Software Inc., San Diego, CA, USA) and SPSS version 17.0 (SPSS Inc., Chicago, IL, USA). For comparisons between two independent groups, the two-tailed Mann–Whitney U test and Chi-square test were applied. Paired data were analyzed using the paired t-test for normally distributed data or the Wilcoxon signed-rank test for non-normally distributed data. For multiple group comparisons, the Kruskal–Wallis H test was used, followed by Bonferroni correction for multiple testing where appropriate. Pearson correlation analysis with the Benjamini–Hochberg correction was performed to evaluate linear associations between continuous variables. Receiver operating characteristic (ROC) analysis was used to evaluate biomarker prognostic performance, and the area under the curve (AUC) with 95% confidence intervals was calculated. Optimal cutoff values were determined using the Youden index and used to stratify patients for survival analysis. Kaplan–Meier curves were compared using the log-rank test. Univariable and multivariable Cox regression models were applied to identify independent mortality predictors, and results are presented as hazard ratios (HRs) with 95% confidence intervals. Data were tested for normality using the Kolmogorov–Smirnov test. Normally distributed variables are presented as mean ± standard deviation, while non-normally distributed variables are reported as median with interquartile range (IQR). A p-value < 0.05 was considered statistically significant.

## Results

### The immune landscape of circulating NK cells in SFTS patients

From previously published scRNA-seq datasets [4], we obtained circulating PBMC samples from SFTS patients and healthy individuals, comprising 4 healthy controls, 11 recovered patients, and 4 deceased patients, totaling 95,739 cells for analysis. NK cells were defined by the positive expression of *NCAM1* or *KLRF1* and the negative expression of *CD3D*, *CD3G*, and *CD3E*, which are highly expressed in effector T cells, NKT cells, and human ILC1s, and were therefore used as negative selection markers to ensure NK cell purity (S1D Fig) [24]. A total of 5,198 NK cells for further analysis were extracted and visualized using a comprehensive Uniform Manifold Approximation and Projection (UMAP) plot, stratified as 1,617 from healthy controls, 2,570 from recovered patients, and 1,011 from deceased patients. NK cells constituted an average of 8.0% of total PBMCs in healthy controls (HC), 4.4% in recovered patients, and 4.7% in deceased patients, with no significant differences in NK cell proportions among groups (S1A, S1B Fig).

Unsupervised clustering analysis of NK cells, guided by canonical markers *NCAM1* (CD56) and *FCGR3A* (CD16), revealed two predominant subsets: CD56^bright^CD16^lo^ and CD56^dim^CD16^hi^ NK cells, corresponding to previously described NK1 and NK2 populations [33]. Each subset exhibited distinct transcriptional signatures characterized by the elevated expression of subset-specific marker genes (Fig 1C; S1 Table). Notably, no adaptive NK cell subset was identified based on *KLRC2* (NKG2C) or *B3GAT1* (CD57) expression (S1D Fig).

Under SFTS infection, the CD56^bright^CD16^lo^ NK cells expanded significantly (Figs 1B, S1E), displaying transcriptional hallmarks of early developmental stages, including high expression of precursor-associated markers such as *SELL, GZMK, IL7R* [33], as shown in Fig 1C. However, compared to the healthy controls, CD56^bright^CD16^lo^ NK cells in SFTS patients exhibited a marked reduction in progenitor-associated genes, such as *IL7R* and *NCAM1*, while showing a significant upregulation of *CD160* and *FCGR3A* (S1C Fig), suggesting that CD56^bright^CD16^lo^ NK cells displayed developmental and functional heterogeneity under SFTS infection. This transcriptional shift suggests that CD56^bright^CD16^lo^ NK cells undergo a maturation trajectory under SFTS disease conditions, shown as Fig 1D, 1E. CD56^bright^CD16^lo^ NK cells exhibited a cytokine-secretory phenotype marked by elevated expression of *GZMK*, *IL18*, *XCL1* and *XCL2* chemokines (Fig 1F).

The CD56^dim^CD16^hi^ NK cells underwent profound phenotypic remodeling during acute SFTS infection. Two clusters (CX3CR1^+^ and PCNA^+^ clusters) were significantly enriched in SFTS patients, all demonstrating hyperactivated cytotoxic profiles with upregulated expression of cytotoxic effector molecules (*GZMA*, *GZMB*, *PRF1*, *NKG7*) as shown in Fig 1F. The CX3CR1^+^ cluster (cytotoxic cluster), representing terminally mature NK cells with maximal *FCGR3A* expression, exhibited the highest cytotoxic potential and upregulated CCL3/CCL4 to recruit adaptive immune cells (Fig 1F). Intriguingly, the CX3CR1^+^ cluster (cytotoxic cluster) also displayed elevated *KLRC2* levels and pronounced upregulation of killer immunoglobulin-like receptors (KIRs), suggesting a more mature developmental trajectory with putative adaptive-like features (Fig 1G). The PCNA^+^ cluster (proliferate cluster) was proliferation-competent, enriched for cell cycle regulators (*STMN1*, *PCNA*, *MKI67*). Conversely, the KLRB1^+^ cluster (cytokine-producing cluster), enriched for cytokine/chemokine genes (*CCL5*, *IFNG*, *IL32*), were depleted during infection(S1E Fig). Pseudotime trajectory and velocity analyses revealed distinct differentiation pathways originating from immature CD56^bright^CD16^lo^ NK cells under healthy and disease conditions, as shown in Fig 1D, 1E. In SFTS, the expansion of CD56^dim^CD16^hi^ NK clusters (CX3CR1^+^ and PCNA^+^ clusters) followed a trajectory distinct from that of KLRB1^+^ cluster, which were predominant in the healthy individuals, suggesting disease-specific NK cell reprogramming.

Collectively, our study delineates a high-resolution transcriptional atlas of circulating NK cells in SFTS infection. These data reveal a dual functional shift in both CD56^bright^ and CD56^dim^ subsets under disease conditions: CD56^bright^CD16^lo^ NK cells are arrested in an immature, cytokine-secretory state, while CD56^dim^CD16^hi^ NK cells undergo cytotoxic hyperactivation coupled with proliferative expansion and subset-specific depletion.

### Upregulation of *HAVCR2* in NK cells of SFTS patients

Previous studies have suggested that NK cells may play a critical role in viral infections such as SFTS [34,35]. To further investigate this, we analyzed the expression of immune checkpoint-related genes in NK cells, including *PDCD1*, *LAG3*, *TIGIT*, and *HAVCR2*. Notably, *LAG3* and *HAVCR2* were significantly upregulated in the SFTS disease group compared to healthy controls (Fig 2A). Furthermore, *HAVCR2* expression exhibited a marked increase in the deceased patients (Deceased vs. Recovered *p* = 0.018, Fig 2A). These findings suggest that TIM-3^+^ NK cells may be associated with poor prognosis in SFTS patients.

To elucidate the functional implications of TIM-3^+^ NK cells, we investigated their transcriptional profile. Differential gene expression analysis revealed that TIM-3^+^ NK cells exhibited upregulation of genes associated with cytotoxicity (*GZMB*, *PRF1*), arachidonic acid metabolism (*PTGDS*), interferon-stimulated responses (*IFI27*, *IFI44L*, *IFI6*, *IFITM3*, *ISG15*), and chemokine signaling (*CCL3*). Conversely, marker genes of CD56^bright^CD16^lo^ NK cells (*GZMK*), immune regulation (*CD52*), and the MAPK signaling pathway (*JUN*) were downregulated (Fig 2B). Enrichment analysis further indicated that TIM-3^+^ NK cells were significantly associated with pathways involved in antiviral immune responses, interferon signaling, and NK cell-mediated cytotoxicity (Fig 2C). These findings suggest that TIM-3^+^ NK cells are functionally regulated by interferons and exhibit enhanced cytotoxic potential. As shown in Fig 2D, 2E, TIM-3^+^ NK cells were distributed across all NK cell subsets, with the CX3CR1^+^ subset (cytotoxic NK cluster) exhibiting the highest average expression and proportion of TIM-3^+^ cells.

Next, we analyzed the expression and distribution of TIM-3 ligands, including *LGALS9* (Galectin-9), *HMGB1*, and *CEACAM1*. Notably, the UMAP plot revealed a considerable overlap in the distribution of cells expressing both *LGALS9* and *HAVCR2*. Cell-cell interaction analysis further revealed that, compared to the recovered group, the deceased group exhibited increased NK cell communication with T cells, dendritic cells, B cells, plasma cells, and monocytes (Fig 2F). Additionally, *LGALS9*-associated signaling pathways were upregulated in NK cells deceased group, including the *LGALS9-HAVCR2* axis. These findings indicate that the TIM-3/Galectin-9 signaling pathway may play a pivotal role in the disease progression of SFTS.

### WGCNA identifies prognosis-associated hub genes in NK cells

Building upon our findings that *HAVCR2* (TIM-3) may be linked to poor prognosis, in order to identify gene co-expression modules associated with poor prognosis at a systematic level, we employed weighted gene correlation network analysis (WGCNA), thereby mitigating the stochastic nature of single-gene analyses.

Network topology analysis classified 27,321 NK cell-associated genes into six distinct modules (Fig 3A). Among them, the turquoise module containing 965 genes was significantly associated with SFTS mortality (Fig 3B, *p* < 0.001). To determine the relevance of each module to poor prognosis, we performed correlation analyses between module membership (MM) and gene significance (GS). The strong correlation between GS and MM indicated that genes highly correlated with clinical traits (GS) were also key constituents of the module (MM). The turquoise module exhibited robust correlation (Cor = 0.94, *p* < 0.001, Fig 3C), suggesting its potential role in disease progression.

To further explore the biological significance of genes within the turquoise module, we constructed a highly interconnected subnetwork comprising the top 48 hub genes in Cytoscape (Fig 3D, Score = 44.17). A second highly connected gene cluster contained 24 genes (Fig 3E, Score = 17.913), including *HAVCR2*, *LAG3*, *TNFSF10* and *TIGIT*. To identify key regulators within this network, we applied MCC scoring using Cytoscape’s cytoHubba plugin and ranked the top ten hub genes, with degree-based color gradients ranging from yellow (low) to red (high). Functional enrichment analysis revealed that the first cluster genes were predominantly associated with interferon responses (Fig 3F), whereas the second cluster genes were enriched in cytotoxic pathways (Fig 3G).

### Increased proportion of circulating TIM-3^+^ NK cells in patients with SFTS

PBMCs were collected from a cohort of 21 patients diagnosed with SFTS, including 17 recovered and 4 deceased individuals in the First Affiliated Hospital of Zhejiang University School of Medicine. 14 healthy donors matched for age and sex were enrolled as controls. Demographic and clinical characteristics of the patients are summarized in Table 1. As previously reported, SFTS was associated with multisystem organ involvement, including pulmonary, hepatic, renal, and central nervous system dysfunction. Compared to recovered patients, deceased patients had significantly higher Charlson Comorbidity Index (CCI) scores (*p* = 0.011), indicating deceased patients were relatively older and had more comorbidities. Deceased patients also showed lower Glasgow Coma Scale (GCS) scores (*p* = 0.013), and significantly elevated viral loads (*p* = 0.002). There were no significant differences in platelet counts, lymphocyte numbers, oxygenation index, or liver and renal function between groups (*p* > 0.050). Notably, the duration of hospitalization was significantly shorter in the deceased group (*p* = 0.003), while the time from symptom onset to admission did not differ (*p* = 0.716), suggesting that rapid early disease progression may be associated with mortality. These findings underscore the urgent need for early prognostic biomarkers and therapeutic targets to improve outcomes in SFTS.

**Fig 4:**
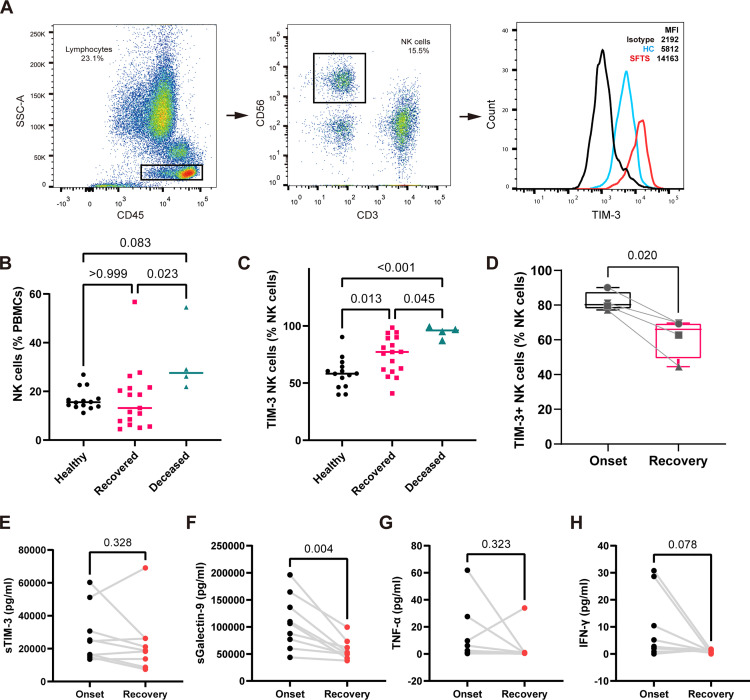
TIM-3 expression on peripheral NK cells in healthy individuals and SFTS patients. **(A)** Representative flow cytometry plots showing TIM-3 expression on NK cells from healthy donors and SFTS patients. **(B)** Frequency of NK cells (CD3 ⁻ CD56⁺) among peripheral blood mononuclear cells (PBMCs) in healthy donors (N = 14), recovered SFTS patients (N = 17), and deceased SFTS patients (N = 4). **(C)** Frequency of TIM-3 ⁺ NK cells among total NK cells in the same cohorts. **(D)** Paired longitudinal analysis of peripheral TIM-3 + NK cell frequencies among NK cells in SFTS patients (N = 4) during the acute phase and approximately two weeks into recovery. Paired longitudinal analysis of soluble TIM-3 **(E)**, Galectin-9 **(F)**, TNF-α **(G)**, IFN-γ **(H)** in plasma of SFTS patients (N = 9) during the acute and recovery phase. Each dot represents an individual donor. Box plots show the full range (min to max). Statistical analysis was performed using Kruskal–Wallis H test for (B); one-way ANOVA with Tukey’s multiple comparisons test for (C) and paired t-test for (D-H). IFN-γ, interferon gamma; NK, natural killer; sGalectin-9, soluble Galectin-9; SFTS, severe fever with thrombocytopenia syndrome; TNF-α, tumor necrosis factor alpha.

Flow cytometric analysis of PBMCs collected at disease onset revealed significant alterations in the proportion of circulating NK cells among PBMCs across groups (Fig 4A, 4B). Patients in the deceased group showed markedly elevated frequencies of NK cells compared to recovered individuals (27.6% [IQR 23.0%–48.1%] vs. 13.2% [IQR 7.1%–21.5%]; *p* = 0.023). Furthermore, the proportion of TIM-3^+^ NK cells among NK cells was significantly increased in the deceased group compared to the recovered group (94.7% ± 5.2% vs. 74.5% ± 16.5%; *p* = 0.045; Fig 4C). Longitudinal analysis of four recovered patients demonstrated a significant decrease in the percentage of TIM-3^+^ NK cells two weeks after symptom onset compared to baseline levels at disease onset (82.0% ± 5.6% vs. 61.6% ± 11.8%; *p* = 0.020; Fig 4D), suggesting a dynamic regulation of TIM-3 expression during recovery. In addition, we assessed the longitudinal changes of serum soluble TIM-3 (sTIM-3) and Galectin-9 concentrations in the same cohort (N = 9; Fig 4E, 4F). Notably, Galectin-9 levels were significantly decreased during recovery (109011 ± 49471 vs. 57348 ± 18960 pg/ml; *p* = 0.004). Cytokines TNF-α and IFN-γ did not show significant longitudinal differences, although IFN-γ levels tended to decline during the recovery phase.

### Enhanced cytotoxic potential of TIM-3^+^ NK cells in SFTS patients

**Fig 5:**
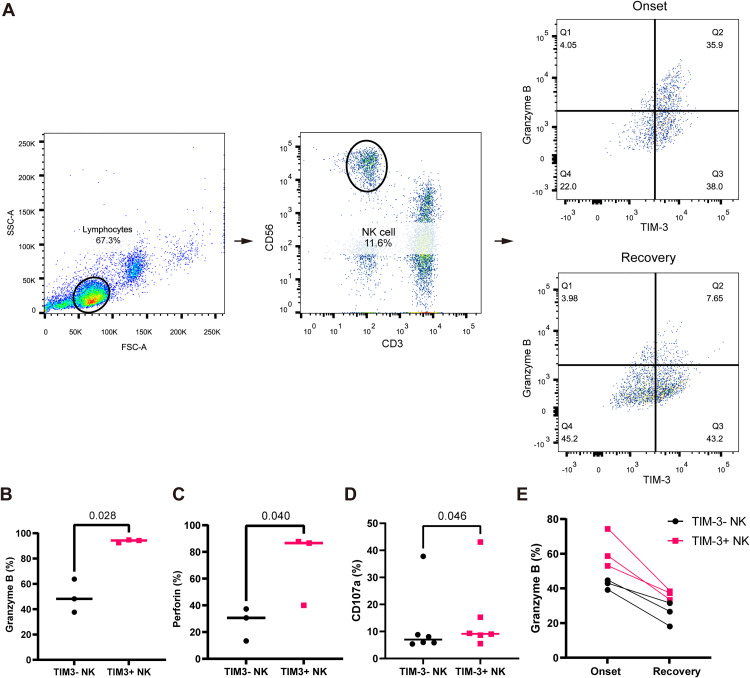
Enhanced cytotoxic potential of TIM-3+ NK cells in SFTS patients. **(A)** Representative flow cytometry plots showing granzyme B expression in TIM-3+ and TIM-3- NK cells isolated from peripheral blood of SFTS patients at disease onset and two weeks into recovery. **(B-D)** Quantification of granzyme B (B), perforin (C) and CD107a (D) expression in TIM-3+ and TIM-3- NK cells at disease onset (N = 3, 3, 6, respectively); median values are indicated. For CD107a detection, PBMCs of SFTS patients at disease onset were stimulated with PMA and ionomycin for 6 hours. **(E)** Paired longitudinal analysis of granzyme B expression in TIM-3+ and TIM-3- NK cells from matched samples at disease onset and recovery phase (N = 3). Statistical analysis was performed using paired t-tests. NK, natural killer; PMA, phorbol 12-myristate 13-acetate; SFTS, severe fever with thrombocytopenia syndrome.

To investigate functional alterations of TIM-3^+^ NK cells in SFTS, paired PBMCs collected at disease onset and two weeks later during recovery were obtained from three patients. NK cells were isolated and stratified based on TIM-3 expression to evaluate cytotoxic marker granzyme B expression (Fig 5A). At disease onset, TIM-3^+^ NK cells exhibited significantly elevated levels of granzyme B, perforin and CD107a compared with their TIM-3 ⁻ counterparts (granzyme B: 93.8% ± 1.2% vs 49.9% ± 13.2%, *p* = 0.028; perforin: 71.4% ± 27.2% vs 27.1% ± 12.3%, *p* = 0.040; CD107a: 15.1% ± 14.0% vs 12.0% ± 12.7%, *p* = 0.046) (Fig 5B–5D). Notably, the proportion of granzyme B ⁺ TIM-3 ⁺ NK cells significantly declined during the recovery phase (*p* = 0.048, Fig 5E), suggesting dynamic modulation of cytotoxic activity in response to disease progression.

### Blockade of TIM-3 attenuates NK cell effector function and cytokine secretion

**Fig 6:**
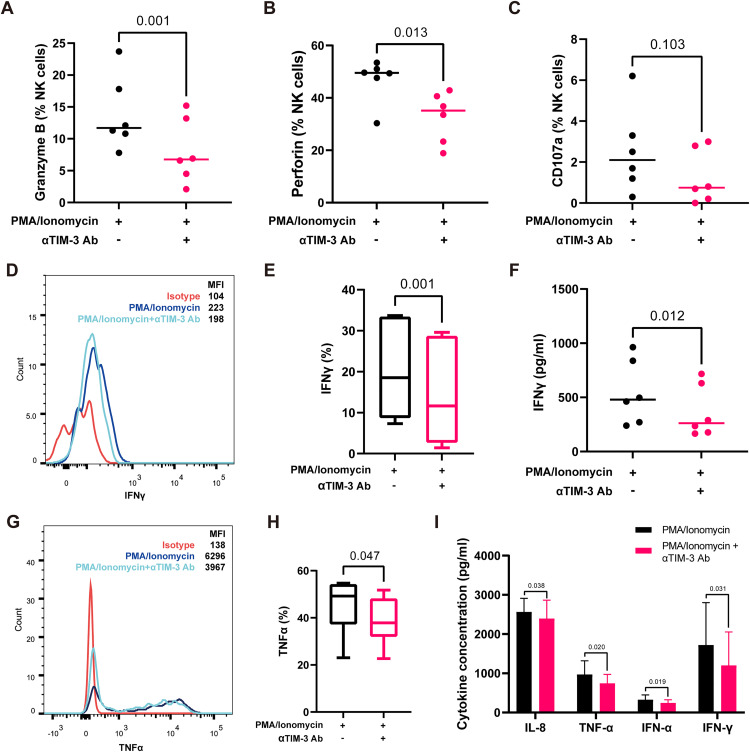
TIM-3 blockade attenuates cytotoxic function of NK cells in SFTS. **(A-C)** Quantification of granzyme B **(A)**, perforin **(B)** and CD107a **(C)** expression in NK cells with and without αTIM-3 antibody treatment after PMA/Ionomycin stimulation (N = 6); median values are indicated. **(D)** Flow cytometric analysis of IFN-γ expression in NK cells following αTIM-3 antibody blockade after PMA/Ionomycin stimulation. **(E)** Quantification of IFN-γ expression in NK cells with or without αTIM-3 antibody treatment (N = 6); box plots show minimum to maximum values. **(F)** Quantification of IFN-γ concentration in NK cells supernatant with and without αTIM-3 antibody treatment after stimulation (N = 6). **(G)** Flow cytometric analysis of TNF-α expression in NK cells following TIM-3 blockade after PMA/Ionomycin stimulation. **(H)** Quantification of TNF-α expression in NK cells with or without αTIM-3 antibody treatment (N = 6); box plots show minimum to maximum values. **(I)** Cytokine concentration in NK cells supernatant with and without αTIM-3 antibody treatment after stimulation using a CBA kit (N = 6); data are presented as mean ± SD. Statistical analysis was performed using paired t-test. αTIM-3 Ab, anti-TIM-3 antibody; CBA, cytometric bead array; IFN-γ, interferon gamma; MFI, mean fluorescence intensity; SD, standard deviation; TNF-α, tumor necrosis factor alpha.

To further assess the role of TIM-3 in NK cell-mediated cytotoxicity, circulating NK cells isolated from SFTS patients were stimulated ex vivo with PMA/ionomycin in the presence or absence of an anti–TIM-3 blocking antibody. After 3 hours of stimulation, the proportions of granzyme B⁺ and perforin⁺ NK cells were both significantly reduced following TIM-3 blockade (granzyme B, 13.9% ± 5.8% vs 8.1% ± 5.1%, *p* = 0.001; perforin, 46.9% ± 8.4% vs 32.7% ± 9.6%, *p* = 0.013, N = 6; Fig 6A, 6B). However, after 6 hours of PMA/ionomycin stimulation, TIM-3 blockade did not significantly alter the proportion of CD107a ⁺ NK cells (2.5% ± 2.1% vs 1.3% ± 1.3%, *p* = 0.103, N = 6, Fig 6C), suggesting that TIM-3 inhibition primarily affects intracellular cytotoxic molecule expression rather than degranulation capacity.

We next assessed the impact of TIM-3 blockade on cytokine production. Intracellular cytokine staining revealed that TIM-3 blockade significantly reduced the proportion of IFN-γ^+^ NK cells (20.1% ± 12.4% vs 14.3% ± 13.6%, *p* = 0.001, N = 6; Fig 6D, 6E), along with a concomitant reduction in TNF-α production (45.4% ± 11.9% vs 38.7% ± 10.1%, *p* = 0.047; Fig 6G, 6H) following 6-hour PMA/ionomycin. Consistently, ELISA quantification of NK cell supernatants showed a marked decrease in IFN-γ concentration after TIM-3 antibody treatment upon stimulation (544.8 ± 296.4 vs 369.1 ± 241.8 pg/ml, *p* = 0.012, N = 6; Fig 6F). Furthermore, multiplex cytokine analysis using CBA (Cytometric Bead Array) demonstrated that TIM-3 blockade led to decreased secretion of several inflammatory mediators, including IL-8, TNF-α, IFN-α, and IFN-γ, upon stimulation (IL-8: 2565.2 ± 347.9 vs 2398.3 ± 465.5 pg/ml, *p* = 0.038; TNF-α: 972.9 ± 344.1 vs 744.1 ± 227.4 pg/ml, *p* = 0.020; IFN-α: 328.6 ± 118.2 vs 246.3 ± 78.5, *p* = 0.019; IFN-γ: 1721.3 ± 1081.9 vs 1201.3 ± 853.7, *p* = 0.031; N = 6; S2 Table), further supporting the inhibitory effect of TIM-3 blockade on NK cell cytokine secretion.

To further explore the functional role of the TIM-3/Galectin-9 axis, we examined the direct effect of Galectin-9 on NK cell cytokine production. Purified NK cells from SFTS patients, pre-activated with IL-12 and IL-18, were stimulated with recombinant Galectin-9. This stimulation significantly increased IFN-γ secretion, an effect that was completely abrogated by the addition of a TIM-3 blocking antibody (S2 Fig). This finding is consistent with previous reports suggesting that Galectin-9 can enhance NK cell effector function via a TIM-3-dependent pathway, and further highlights the complex, potentially activating role of this axis in SFTS immunopathology.

### Identification of sTIM-3 and sGalectin-9 as prognostic serum biomarkers in SFTS

**Fig 7:**
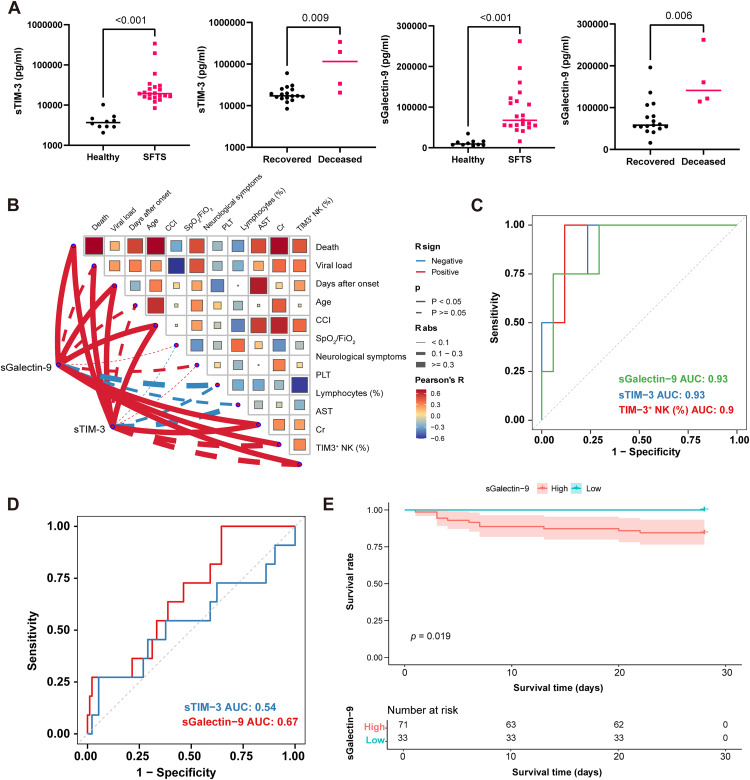
Soluble TIM-3 and Galectin-9 as serum biomarkers for SFTS prognosis. **(A)** Serum levels of soluble TIM-3 (sTIM-3) and Galectin-9 in healthy individuals (N = 10), recovered SFTS patients (N = 17), and deceased SFTS patients (N = 4). **(B)** Pearson correlation analysis between sTIM-3 levels, Galectin-9 levels, and the frequency of TIM-3^+^ NK cells among NK cells with clinical characteristics. **(C)** ROC curves of serum Galectin-9, sTIM-3, and the frequency of TIM-3 ⁺ NK cells in predicting fatal outcome in the same cohort. **(D)** ROC curves of serum Galectin-9 and sTIM-3 in the validation cohort (N = 104), with AUC, sensitivity, and specificity calculated using the Youden index. **(E)** Kaplan–Meier survival analysis stratified by high vs. low serum Galectin-9 levels in the validation cohort (N = 104), using the ROC-derived optimal cutoff. Statistical significance was assessed using the Mann-Whitney test for (A), and Pearson correlation analysis with the Benjamini-Hochberg correction for (B), DeLong’s test for (C-D) and the log-rank test for (E). sTIM-3, soluble T cell immunoglobulin and mucin domain-3; Gal-9, galectin-9; CCI, Charlson comorbidity index; PLT, platelet count; AST, aspartate aminotransferase; Cr, creatinine; ROC, receiver operating characteristic curve; AUC, area under the ROC curve.

Building upon previous findings, we further investigated TIM-3-related soluble factors as potential prognostic biomarkers in SFTS. Serum levels of sTIM-3 and sGalectin-9 were measured in healthy individuals and in patients with SFTS. Both sTIM-3 and sGalectin-9 were significantly elevated in the serum of SFTS patients compared to healthy controls (Fig 7A; *p* < 0.001). Deceased patients exhibited significant higher levels of sTIM-3 and sGalectin-9 compared to recovered individuals (Fig 7A; sTIM-3, *p* = 0.009; sGalectin-9, *p* = 0.006).

We next performed Pearson correlation analyses to explore the relationships between serum sTIM-3, sGalectin-9, the proportion of TIM-3 ⁺ NK cells, and clinical parameters (Fig 7B). Notably, sTIM-3 levels were positively correlated with fatal outcome (r = 0.65, *p* = 0.001), viral load (r = 0.50, *p* = 0.020), and aspartate aminotransferase (AST) levels (r = 0.86, *p* < 0.001). sGalectin-9 also showed a significant correlation with the fatal outcome (r = 0.62, *p* = 0.003), TIM-3^+^ NK proportion (r = 0.52, *p* = 0.017), aspartate aminotransferase (AST) levels (r = 0.69, *p* = 0.001) and creatine levels (r = 0.62, *p* = 0.003). Additionally, the proportion of TIM-3^+^ NK cells was correlated with the fatal outcome (r = 0.46, *p* = 0.033), and inversely correlated with platelet count (r = –0.58, *p* = 0.006), suggesting a potential link between TIM-3^+^ NK cell expansion and hematologic dysregulation. In the discovery cohort (N = 21), receiver operating characteristic (ROC) analysis showed that serum Galectin-9, sTIM-3, and the frequency of TIM-3 ⁺ NK cells achieved area under the ROC curve (AUC) values of 0.927 (95% CI: 0.812–1.000), 0.927 (95% CI: 0.797–1.000), and 0.897 (95% CI: 0.738–1.000), respectively (Fig 7C).

To validate these findings, an independent cohort of 104 confirmed SFTS patients from Nanjing Drum Tower Hospital was analyzed. Serum Galectin-9 and sTIM-3 yielded AUCs of 0.671 (95% CI: 0.513–0.828) and 0.542 (95% CI: 0.324–0.759), respectively, indicating that Galectin-9 had superior prognostic performance compared with sTIM-3 (Fig 7D). Based on the optimal cutoff derived from the Galectin-9 ROC curve, patients were stratified into high- and low-expression groups. Kaplan–Meier analysis revealed significantly different 28-day survival outcomes between the two groups (Fig 7E, *p* = 0.019). To further assess the prognostic value of serum markers, univariable and multivariable Cox regression analyses were performed. Galectin-9 remained significantly associated with mortality after adjustment for age, sex, and Charlson comorbidity index (adjusted *p* = 0.004), whereas sTIM-3 showed no significant association with survival (S4 Table). These findings support serum Galectin-9, better than sTIM-3, as circulating prognostic biomarkers in SFTS, warranting validation in larger prospective cohorts with multivariate analyses.

## Discussion

In this study, we systematically delineated the peripheral NK cell immune landscape in patients with SFTS and revealed the critical role of TIM-3 ⁺ NK cells in disease progression. Through integration of single-cell RNA sequencing, WGCNA, flow cytometry, functional assays, and serological analyses, we demonstrated that elevated TIM-3 expression is closely associated with enhanced NK cell cytotoxicity and elevated production of inflammatory cytokines. Functional blockade of TIM-3 attenuated NK cell effector responses, supporting a role for TIM-3 signaling in infection-associated NK cell activation. Notably, both soluble TIM-3 and Galectin-9 were markedly increased in patients with severe disease, and serum Galectin-9—validated in an independent cohort—demonstrated significant prognostic value for 28-day mortality. These findings delineate a TIM-3–associated NK cell hyperactivation axis in SFTS and highlight Galectin-9 as a promising circulating biomarker for early risk stratification.

Previous studies on SFTS have primarily focused on cytokine storms, T cell dysregulation and monocyte–macrophage dysfunction. B cells, particularly plasma cells, have also been implicated as potential viral reservoirs in patients with fatal outcomes [36]. In contrast, the role of NK cells in SFTS remains relatively understudied, and existing findings are often inconsistent. One study involving 29 patients reported that, although CD56^dim^CD16^hi^ NK cells were depleted during acute infection, they exhibited enhanced activation, as indicated by increased expression of Ki-67 and granzyme B, along with reduced levels of the inhibitory receptor NKG2A, suggesting their potential involvement in early antiviral responses [21]. Conversely, another study of 34 patients observed an overall increase in peripheral NK cell frequencies, particularly during the acute and severe phases of the disease [22]. Such inconsistencies highlight a critical knowledge gap regarding NK cell dynamics during SFTS progression.

Monitoring peripheral NK cell frequency and function provides valuable insight into host immune responses during viral infection. In acute viral infections, circulating conventional NK (cNK) cells are recruited to infected tissues, where they differentiate into tissue-resident NK (trNK) cells [37]. Thus, alterations in peripheral NK cell composition may both reflect and contribute to local immune responses. Similar redistribution and phenotypic remodeling of NK cells have been observed in several viral infection models, including human immunodeficiency virus (HIV), cytomegalovirus (CMV) and SARS-CoV-2, where dynamic shifts in NK subset frequencies correlate with activation or exhaustion states at infection sites [38]. Therefore, characterizing peripheral NK cell subsets—particularly the proportion and function of circulating TIM-3 ⁺ NK cells—offers a useful window into the immunopathogenesis of severe SFTS. The elevated frequency and enhanced cytotoxic and cytokine-secreting capacity of TIM-3 ⁺ NK cells in severe SFTS patients likely indicate immune dysregulation, reflecting excessive NK activation and migration at sites of infection.

Our findings revealed that TIM-3^+^ NK cells exhibit enhanced cytotoxic potential yet associated with disease severity and poor prognosis, suggesting that excessive NK cell activation may contribute to immunopathology, such as cytokine storms or tissue damage. In healthy individuals, TIM-3 has been described as a marker of mature NK cells, consistent with our observations [17]. Reports from other infections, including COVID-19, have also documented aberrant NK cell phenotypes [14]. In acute myeloid leukemia, elevated TIM-3 expression on NK cells correlates positively with perforin and granzyme B levels and is associated with improved clinical outcomes [39]. In human papillomavirus-positive HNSCC, TIM-3^+^ NK cells demonstrate heightened effector potential, and their function is inhibited by Galectin-9 via TIM-3 signaling [20]. In our study, TIM-3^+^ NK cells exhibited enhanced cytotoxic function, and their activity was reduced upon TIM-3 antibody blockade, suggesting that TIM-3 may play a context-dependent, non-canonical regulatory role rather than acting solely as an inhibitory checkpoint. Collectively, these findings, along with previous reports, support a dual role of TIM-3, with its functional outcomes being highly dependent on cell type and disease context. As this represents a non-canonical role of TIM-3, few studies have explored its regulatory mechanisms in NK cells. High TIM-3 expression may promote cytokine release such as IFN-γ, and engagement with Galectin-9 has been shown to enhance IFN-γ production in a TIM-3–dependent manner [40]. These findings suggest that TIM-3 signaling can augment NK cell proinflammatory activity under certain conditions, with its functional outcome largely dependent on the nature of the activating stimuli [41].

SFTS is marked by profound immune dysregulation, yet the role of immune checkpoint molecules in this process has been insufficiently explored. Recent insights highlight the importance of immune checkpoint pathways in modulating antiviral immunity. The regulation of TIM-3 ⁺ NK cells appears to involve cooperative interactions with multiple receptors. In some contexts, such as chronic infection or cancer, TIM-3 ⁺ NK cells co-express other inhibitory receptors, such as PD-1, suggesting a cooperative mechanism that drives NK cell exhaustion [42]. Similar phenotypes are observed in other disease contexts, such as HNSCC, where TIM-3 ⁺ NK cells often co-express CD44, a marker associated with hyperactivated effector function [20]. Additional evidence supporting the involvement of immune checkpoints in SFTS pathogenesis includes the observation that PD-1 blockade in vitro effectively suppresses SFTSV replication [43]. Moreover, SFTS patients display a highly exhausted CD8 ⁺ T cell phenotype, which is associated with persistent viral infection and poor clinical outcomes [4].

Despite these advances in understanding immune dysfunction, effective immunotherapeutic strategies for SFTS remain limited. Although intravenous immunoglobulin is widely used in the treatment of various viral infections, it has shown minimal benefit in improving clinical outcomes in SFTS [34]. In contrast, targeted immunomodulatory therapies offer more promise. Ruxolitinib, a Janus kinase 1/2 inhibitor, effectively dampens pro-inflammatory cytokine signaling and suppresses type I interferon pathways, leading to significantly reduced 28-day mortality in clinical studies. Similarly, the IL-6 receptor antagonist tocilizumab has shown comparable clinical benefit [9,44].

Efforts to identify prognostic indicators for SFTS have incorporated diverse biological parameters, including immune dysregulation, inflammatory status, coagulopathy, and degree of organ injury. A retrospective study involving 96 patients identified advanced age, elevated IL-6 levels, and reduced CD4 ⁺ T cell counts as predictors of disease severity [45]. In a larger prospective cohort of 714 patients, a prognostic scoring model was established based on age, admission temperature, leukocyte and platelet counts, AST, serum creatinine, and vasopressor use [46]. A machine learning model developed from 1,606 cases further integrated viral load, activated partial thromboplastin time, AST, disturbance of consciousness, blood urea nitrogen, and age to accurately predict mortality risk [47]. Nonetheless, reliable early biomarkers for risk stratification and prognostication remain elusive. This underscores the urgent need for novel and robust immune-based biomarkers that reflect host-pathogen interactions and disease trajectory.

In this study, we provide initial evidence that TIM-3 ⁺ NK cells may serve as potential prognostic indicators in SFTS. In parallel, sTIM-3 and sGalectin-9 levels emerged as candidate serum biomarkers of disease severity. However, our findings are constrained by the limited sample size, retrospective single-center design, and small number of fatal cases, which may limit statistical power and generalizability. Because of the limited number of subjects, regression analyses could not be reliably performed; therefore, potential confounding effects of age and comorbidities cannot be fully excluded. Further validation in larger, multicenter prospective cohorts is essential. Additionally, while our analysis identifies infection-associated reprogramming of NK cells and establishes clinically relevant correlations, the underlying mechanisms remain incompletely defined, and a causal relationship between TIM-3 signaling and NK-cell hyperactivation has not yet been demonstrated. Future investigations using animal models and in vitro co-culture systems will be important to delineate the functional role of TIM-3 ⁺ NK cells in SFTS immunopathology. Moreover, our single-cell analyses were performed only at the acute stage of infection. Longitudinal profiling—including parallel assessment of NK-cell states in acute and convalescent phases—will be essential to validate the dynamic transitions inferred from trajectory analysis and to reduce inter-individual variability. Ultimately, unraveling the immune checkpoint landscape in SFTS may not only enhance prognostic accuracy but also inform the development of novel immunomodulatory strategies for acute viral infection.

## Supporting information

S1 Table Top10 marker genes of NK cell subclusters. (DOCX)

S2 Table Cytokine concentrations in NK cell supernatants following TIM-3 blockade. (DOCX)

S3 Table Baseline characteristics of SFTS patients in the validation cohort (N = 104). (DOCX)

S4 Table Cox regression analysis of serum Galectin-9 and soluble TIM-3 levels associated with mortality in the validation cohort (N = 104). (DOCX)

S1 Fig Characterization of NK cell subclusters in SFTS patients. (A) UMAP projection of NK cells from healthy controls (HC), recovered patients, and deceased patients. (B) Proportions of NK cells among total PBMCs show no significant differences across groups. (C) UMAP feature plots showing the expression of *KLRC2* and *B3GAT1*, markers of adaptive NK cells. (D) Differential expression of genes in CD56^bright^CD16^lo^ NK cells from SFTS patients versus healthy controls. (E) Relative abundance of NK subclusters across clinical groups. Statistical significance was assessed using unpaired two-tailed t-tests. NK, natural killer; HC, healthy controls; SFTS, severe fever with thrombocytopenia syndrome.
(DOCX)

S2 Fig Effects of Galectin-9 on IFN-γ secretion by NK cells from SFTS patients and its modulation by TIM-3 blockade. Purified NK cells from SFTS patients were pre-activated with IL-12 (1 ng/mL) and IL-18 (10 ng/mL) for 16 hours, followed by treatment with recombinant human Galectin-9 (20 nM) alone or in combination with anti–TIM-3 blocking antibody. Supernatants were collected for quantification of IFN-γ concentration (N = 4). Statistical analysis was performed using paired one-way ANOVA with Tukey’s multiple-comparison test. αTIM-3 Ab, anti–TIM-3 antibody; IFN-γ, interferon gamma.
(DOCX)
