# Supplementary material for: Immune checkpoint TIM-3 defines hyperactivated NK cells and predicts fatal outcome in severe fever with thrombocytopenia syndrome
Source: PLoS Negl Trop Dis. 2026 Jan 16;20(1):e0013928. doi: 10.1371/journal.pntd.0013928 (PMC12829940; doi:10.1371/journal.pntd.0013928)
Supplement: S1 Table — (DOCX) [file pntd.0013928.s001.docx]

**S1 Table. Top10 marker genes of NK cell subclusters.**

| **CD56^bright^CD16^lo^** | **CD56^dim^CD16^hi^**  **c1_CX3CR1** | **CD56^dim^CD16^hi^**  **c2_KLRB1** | **CD56^dim^CD16^hi^**  **c3_PCNA** |
| --- | --- | --- | --- |
| AREG | ISG15 | KLRB1 | STMN1 |
| SELL | IFIT3 | DUSP2 | DUT |
| IFITM3 | XAF1 | JUN | TUBA1B |
| COTL1 | CX3CR1 | JUNB | TYMS |
| IL2RB | IFIT1 | CD52 | PCNA |
| GZMK | RSAD2 | JUND | MCM5 |
| KLRC1 | LAG3 | IER2 | MCM7 |
| CAPG | PTGDS | NR4A2 | CLSPN |
| IL7R | HBA1 | SNHG5 | PCLAF |
| IFI27 | HBA2 | CXCR4 | HMGB2 |
