# Supplementary material for: Immune checkpoint TIM-3 defines hyperactivated NK cells and predicts fatal outcome in severe fever with thrombocytopenia syndrome
Source: PLoS Negl Trop Dis. 2026 Jan 16;20(1):e0013928. doi: 10.1371/journal.pntd.0013928 (PMC12829940; doi:10.1371/journal.pntd.0013928)
Supplement: S2 Table — (DOCX) [file pntd.0013928.s002.docx]

**S2 Table. Cytokine concentrations in NK cell supernatants following TIM-3 blockade.**

| **Cytokines** | **Supernatant concentration (pg/ml)** | | ***p* value** |
| --- | --- | --- | --- |
|  | PMA/Ionomycin stimulation | PMA/Ionomycin + αTIM-3 Ab |  |
| IFN-α | 328.6 ± 118.2 | 246.3 ± 78.5 | **0.019** |
| IFN-γ | 1721.3 ± 1081.9 | 1201.3 ± 853.7 | **0.031** |
| IL-10 | 5.4 ± 1.8 | 5.2 ± 2.0 | 0.542 |
| IL-12p70 | 11.5 ± 1.9 | 10.8 ± 2.1 | 0.290 |
| IL-17A | 4.6 ± 1.0 | 5.3 ± 3.2 | 0.655 |
| IL-1β | 127.0 ± 96.2 | 130.3 ± 102.4 | 0.814 |
| IL-2 | 63.1 ± 35.2 | 43.4 ± 25.1 | 0.174 |
| IL-4 | 3.9 ± 1.0 | 3.6 ± 1.0 | 0.113 |
| IL-5 | 2.0 ± 0.3 | 2.0 ± 0.1 | 0.636 |
| IL-6 | 41.1 ± 29.9 | 48.3 ± 35.7 | **0.045** |
| IL-8 | 2565.2 ± 347.9 | 2398.3 ± 465.5 | **0.038** |
| TNF-α | 972.9 ± 344.1 | 744.1 ± 227.4 | **0.020** |

Cytokine levels were measured in NK cell culture supernatants with or without αTIM-3 antibody treatment after PMA/Ionomycin stimulation, using a CBA kit (N = 6). Data are presented as mean ± SD. Statistical analysis was performed using a paired t-test. αTIM-3 Ab, anti–TIM-3 antibody.
