# Supplementary material for: Immune checkpoint TIM-3 defines hyperactivated NK cells and predicts fatal outcome in severe fever with thrombocytopenia syndrome
Source: PLoS Negl Trop Dis. 2026 Jan 16;20(1):e0013928. doi: 10.1371/journal.pntd.0013928 (PMC12829940; doi:10.1371/journal.pntd.0013928)
Supplement: S3 Table — (DOCX) [file pntd.0013928.s003.docx]

**S3 Table.** **Baseline characteristics of SFTS patients in the validation cohort (N = 104).**

|  | **Total (N=104)** | **Recovered (N=93)** | **Deceased (N=11)** | ***P* Value** |
| --- | --- | --- | --- | --- |
| **Demography** | | | | |
| Age | 62.0 (54.0 - 71.3) | 62.0 (54.0 - 71.0) | 69.0 (57.5 - 75.0) | 0.249 |
| Male Sex (n,%) | 42 (40.4) | 38 (36.5) | 4 (3.8) | 0.522 |
| Neurological symptom | 25 (24.0) | 14 (13.5) | 11 (10.6) | **<0.001** |
| Days from symptom onset to hospital admission (day) | 8.5 (5.0 - 11.0) | 9.0 (5.0 - 11.0) | 8.0 (5.5 - 12.0) | 0.552 |
| Hospitalization period (day) | 9.0 (6.0 - 12.3) | 9.0 (7.0 - 12.0) | 6.0 (3.0 - 10.0) | 0.181 |
| **Comorbidity** | | | | |
| Hypertension (n,%) | 27 (26.0) | 25 (24.0) | 2 (1.9) | 0.717 |
| Diabetes (n,%) | 13 (12.5) | 12 (11.5) | 1 (1.0) | 0.587 |
| Cancer (n,%) | 4 (3.8) | 4 (3.8) | 0 (0.0) | 0.635 |
| CCI (median [IQR]) | 2.0 (1.0 - 3.0) | 2.0 (1.0 - 3.0) | 3.0 (1.5 - 3.0) | 0.353 |
| **Laboratory parameters** | | | | |
| WBC (10^9^/L) | 2.4 (1.8 - 3.4) | 2.8 (1.8 - 3.4) | 2.0 (1.9 - 2.9) | 0.638 |
| PLT (10^9^/L) | 64.0 (42.8 - 91.5) | 64.0 (42.8 - 91.5) | 63.5 (45.3 - 78.3) | 0.230 |
| Lymphocyte (%) | 24.3 (16.1 - 39.1) | 24.3 (16.0 - 39.1) | 27.8 (21.1 - 36.1) | 0.771 |
| ALT (U/L) | 55.5 (37.0 - 97.5) | 53.0 (36.3 - 83.5) | 100.0 (79.5 - 227.0) | 0.139 |
| AST (U/L) | 125.0 (72.3 - 239.2) | 114.8 (70.3 - 199.4) | 398.0 (366.3 - 439.5) | **0.012** |
| Cr (μmol/L) | 80.0 (65.3 - 99.0) | 79.6 (64.1 - 99.7) | 80.0 (69.6 - 82.9) | 0.177 |

Data are presented as median [interquartile range] or n (%), as appropriate. Statistical analysis was performed using the Mann-Whitney test and Chi-square test. CCI, Charlson comorbidity index; WBC, white blood cell count; PLT, platelet count; ALT, alanine aminotransferase; AST, aspartate aminotransferase; Cr, creatinine; SFTS, severe fever with thrombocytopenia syndrome; NA, not available.
