# Supplementary material for: Immune checkpoint TIM-3 defines hyperactivated NK cells and predicts fatal outcome in severe fever with thrombocytopenia syndrome
Source: PLoS Negl Trop Dis. 2026 Jan 16;20(1):e0013928. doi: 10.1371/journal.pntd.0013928 (PMC12829940; doi:10.1371/journal.pntd.0013928)
Supplement: S4 Table — (DOCX) [file pntd.0013928.s004.docx]

**S4 Table. Cox regression analysis of serum Galectin-9 and soluble TIM-3 levels associated with mortality in the validation cohort (N = 104).**

|  | **Variables** | **aHR** | ***P*** |
| --- | --- | --- | --- |
| **Model 1** | sGalectin-9 | 1.00 (1.00 - 1.00) | **0.002** |
|  | sTIM-3 | 1.00 (1.00 - 1.00) | 0.266 |
| **Model 2** | sGalectin-9 | 1.00 (1.00 - 1.00) | **0.003** |
|  | sTIM-3 | 1.00 (1.00 - 1.00) | 0.210 |
| **Model 3** | sGalectin-9 | 1.00 (1.00 - 1.00) | **0.004** |
|  | sTIM-3 | 1.00 (1.00 - 1.00) | 0.215 |

Cox proportional hazards regression was applied to evaluate the association between serum biomarkers and mortality. Model 1 represents the unadjusted analysis; Model 2 is adjusted for age and sex; and Model 3 is adjusted for age, sex, and Charlson Comorbidity Index (CCI). Abbreviations: aHR, adjusted hazard ratio; CI, confidence interval; sGalectin-9, soluble Galectin-9; sTIM-3, soluble T cell immunoglobulin and mucin domain-3; CCI, Charlson Comorbidity Index.
