# Supplementary material for: Immune checkpoint TIM-3 defines hyperactivated NK cells and predicts fatal outcome in severe fever with thrombocytopenia syndrome
Source: PLoS Negl Trop Dis. 2026 Jan 16;20(1):e0013928. doi: 10.1371/journal.pntd.0013928 (PMC12829940; doi:10.1371/journal.pntd.0013928)
Supplement: S1 Fig — (A) UMAP projection of NK cells from healthy controls (HC), recovered patients, and deceased patients. (B) Proportions of NK cells among total PBMCs show no significant differences across groups. (C) UMAP feature plots showing the expression of KLRC2 and B3GAT1, markers of adaptive NK cells. (D) Differential expression of genes in CD56brightCD16lo NK cells from SFTS patients versus healthy controls. (E) Relative abundance of NK subclusters across clinical groups. Statistical significance was assessed using unpaired two-tailed t-tests. NK, natural killer; HC, healthy controls; SFTS, severe fever with thrombocytopenia syndrome. (DOCX) [file pntd.0013928.s005.docx]

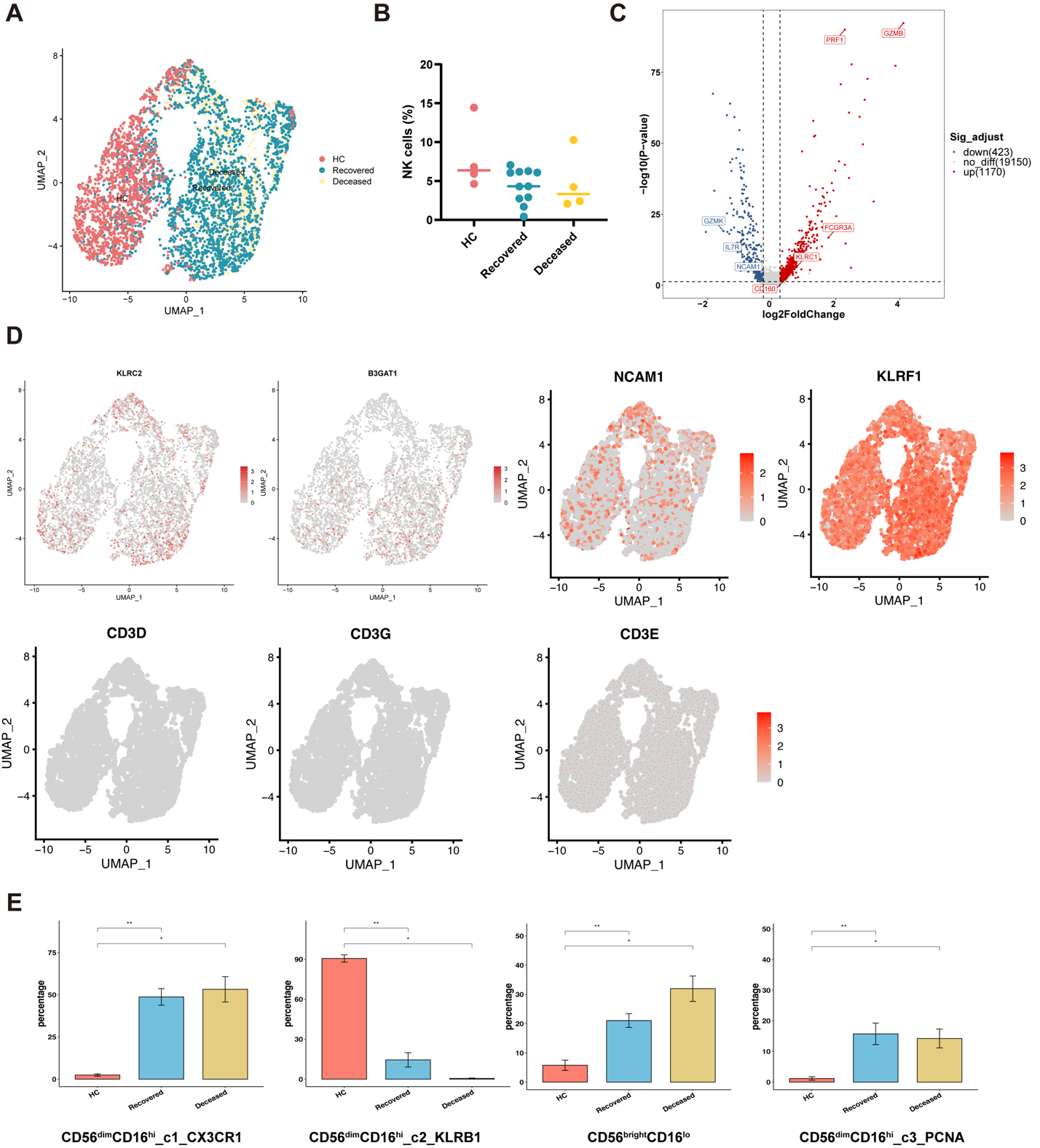


**S1 Fig. Characterization of NK cell subclusters in SFTS patients.** (A) UMAP projection of NK cells from healthy controls (HC), recovered patients, and deceased patients. (B) Proportions of NK cells among total PBMCs show no significant differences across groups. (C) Differential expression of genes in CD56^bright^CD16^lo^ NK cells from SFTS patients versus healthy controls. (D) UMAP feature plots showing the expression of NK cell–identifying markers (NCAM1, KLRF1, CD3D, CD3G, CD3E) and adaptive NK cell–associated markers (KLRC2 and B3GAT1). (E) Relative abundance of NK subclusters across clinical groups. Statistical significance was assessed using unpaired two-tailed t-tests. NK, natural killer; HC, healthy controls; SFTS, severe fever with thrombocytopenia syndrome.
