# Supplementary material for: Immune checkpoint TIM-3 defines hyperactivated NK cells and predicts fatal outcome in severe fever with thrombocytopenia syndrome
Source: PLoS Negl Trop Dis. 2026 Jan 16;20(1):e0013928. doi: 10.1371/journal.pntd.0013928 (PMC12829940; doi:10.1371/journal.pntd.0013928)
Supplement: S2 Fig — Purified NK cells from SFTS patients were pre-activated with IL-12 (1 ng/mL) and IL-18 (10 ng/mL) for 16 hours, followed by treatment with recombinant human Galectin-9 (20 nM) alone or in combination with anti–TIM-3 blocking antibody. Supernatants were collected for quantification of IFN-γ concentration (N = 4). Statistical analysis was performed using paired one-way ANOVA with Tukey’s multiple-comparison test. αTIM-3 Ab, anti–TIM-3 antibody; IFN-γ, interferon gamma. (DOCX) [file pntd.0013928.s006.docx]

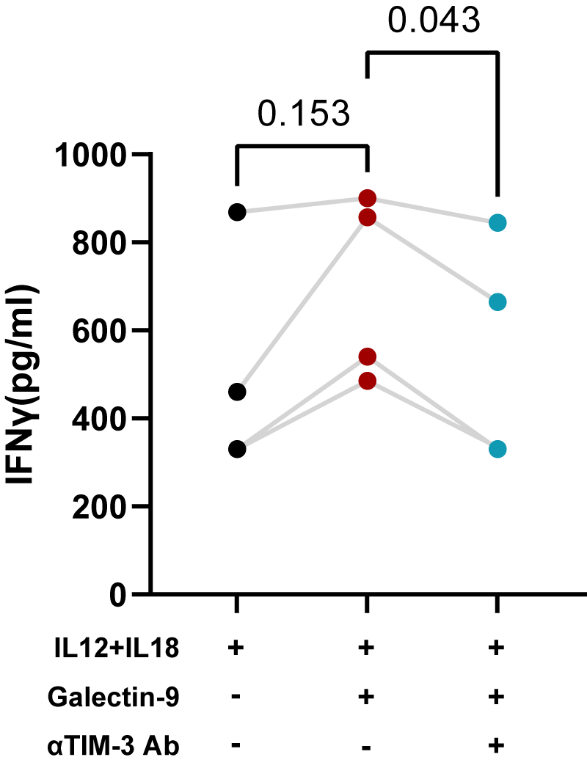


**S2 Fig. Effects of Galectin-9 on IFN-γ secretion by NK cells from SFTS patients and its modulation by TIM-3 blockade.**

Purified NK cells from SFTS patients were pre-activated with IL-12 (1 ng/mL) and IL-18 (10 ng/mL) for 16 hours, followed by treatment with recombinant human Galectin-9 (20 nM) alone or in combination with anti–TIM-3 blocking antibody. Supernatants were collected for quantification of IFN-γ concentration (N = 4). Statistical analysis was performed using paired one-way ANOVA with Tukey’s multiple-comparison test. αTIM-3 Ab, anti–TIM-3 antibody; IFN-γ, interferon gamma.
